# Bolstering agreement with scarce resource allocation policy using education: a post hoc analysis of a randomized controlled trial

**DOI:** 10.1186/s12913-025-12712-x

**Published:** 2025-04-14

**Authors:** Russell G. Buhr, Cher X. Huang, Ruby Romero, Lauren E. Wisk

**Affiliations:** 1 Division of Pulmonary and Critical Care Medicine, David Geffen School of Medicine at the University of California, Los Angeles, 1100 Glendon Avenue, Suite 850, Los Angeles, CA 90024 USA; 2 Center for the Study of Healthcare Innovation, Implementation, and Policy, Health Services Research, Greater Los Angeles Veterans Affairs Healthcare System, Los Angeles, CA USA; 3 Division of General Internal Medicine and Health Services Research, David Geffen School of Medicine at the University of California, Los Angeles, Los Angeles, CA USA; 4 Department of Health Policy and Management, Fielding School of Public Health at the University of California, Los Angeles, Los Angeles, CA USA

**Keywords:** Scarce resource allocation, Crisis standards of care, Patient education, Internet-based survey, Critical care

## Abstract

**Background:**

The COVID- 19 pandemic prompted rapid development of scarce resource allocation policies (SRAP) in case demand for critical health services eclipsed capacity. We sought to test whether a brief, educational video could improve alignment of participant values and preferences with the tenets of the University of California Health’s SRAP in a post hoc analysis of a randomized controlled trial (RCT) conducted during the pandemic.

**Methods:**

An RCT of an educational video intervention embedded in a longitudinal web-based survey conducted from May to December 2020, analyzed in August 2024. The “explainer” video intervention was approximately 6 min long and provided an overview of the mechanics and ethical principles underpinning the UC Health SRAP, subtitled in six languages. California residents were randomized to view the intervention or not, stratified by age, sex, education, racial identity, and self-reported health care worker status. Non-California residents were assigned to the control group. 1,971 adult participants were enrolled at baseline, and 939 completed follow-up. 770 participants with matched baseline and follow-up responses were analyzed. Self-reported survey assessments of values regarding components of SRAP were scored as the percentage of agreement with the UC Health SRAP as written. Participants responded to items at baseline and follow-up (approximately 10 weeks after baseline), with randomization occurring between administrations.

**Results:**

After the intervention, overall agreement improved by a substantial margin of 5.2% (from 3.8% to 6.6%, *P* <.001) for the intervention group compared to the control group. Significant changes in agreement with SRAP logistics and health factors were also observed in the intervention group relative to the control, while no significant changes were noted for social factors. Differential intervention effects were observed for certain demographic subgroups.

**Conclusions:**

A brief educational video effectively explains the complex ethical principles and mechanisms of the SRAP, as well as how to improve the alignment of participant values with the foundational principles of UC Health SRAP. This directly informs practice by providing a framework for educating individuals about the use of these policies during future situations that require crisis standards of care, which can, in turn, enhance agreement and buy-in from affected parties.

**Trial registration:**

ClinicalTrials.gov registration NCT04373135 (registered 4 May 2020).

**Supplementary Information:**

The online version contains supplementary material available at 10.1186/s12913-025-12712-x.

## Key points

**Question:**

Can a brief educational video improve agreement with the ethical principles underlying scarce resource allocation policies (SRAP)?

**Findings:**

In this post hoc analysis of a randomized controlled trial, we observed that overall agreement with SRAP improved by a significantly greater margin of 5.2% (3.8% to 6.6%, P <.001) for intervention relative to control. Effect heterogeneity was seen for some demographic subgroups.

**Meaning:**

Educational interventions are effective for nudging alignment of personal values with ethical principles while observed effect heterogeneity highlights the need for additional research to tailor and target messaging to maximize buy-in.

**Supplementary Information:**

The online version contains supplementary material available at 10.1186/s12913-025-12712-x.

## Introduction

Scarce resource allocation policies (SRAP) outline processes by which limited resources, such as mechanical ventilators, are allocated during critical care shortages [1, 2]. During the COVID-19 pandemic, many policies were designed with limited community engagement due to the emergent nature of the rapidly worsening crisis and difficulty recruiting and convening advisors outside of the working group during active stay-at-home orders [3–6]. Having designed such a policy in 2020, the University of California (UC) [7] chartered the Understanding Community Considerations, Opinions, Values, Impacts, and Decisions (UC-COVID) study to seek public opinion on draft UC SRAP rapidly [8]. We previously demonstrated moderately high (67% to 83% by domain) community agreement with SRAP tenets across domains of logistical concerns (how SRAP would be implemented), health factors (how a patient’s current and historic health status would affect allocation decisions), social factors (how factors unrelated to health, such as age, would be incorporated), and exceptions (situations where SRAP may be temporarily deferred or exempted) [9].

We also embedded a clinical trial within the UC-COVID study to test the impact of a video intervention on knowledge and trust in SRAP, finding improved community-level SRAP understanding [10]. However, less is known about whether these interventions can enhance agreement with SRAP, of critical importance during emergencies such as the pandemic, as health policy decisions and the authorities that promulgate them can be met with distrust or disagreement, exacerbated by poor communication of the underlying rationale for what can be viewed as a heavy-handed or top-down decision [11–13]. While knowledge about the policy was the principal outcome of our previous study, during the course of our analyses, we formed a secondary research question about whether such an intervention could improve agreement and buy-in with such policies, and if so, among which groups we could affect the most improvement. Here, we present an additional post hoc analysis of the UC-COVID trial to evaluate the impact of an educational video on agreement with SRAP tenets and frameworks.

This analysis offers an empirical test of the efficacy of an explanatory video intervention for this use case but also has implications that extend beyond SRAP to other policy issues where improving key interested party knowledge, trust, or agreement in a potentially controversial policy element is a goal. Such tools would be useful when key informants may not be readily available for the timely collection of input in the design of a complex health service intervention. Additionally, they offer an opportunity to provide rapid, easily disseminable explanations to interested parties who would be affected by such an intervention. As such, the knowledge gained in this study is applicable beyond the use for SRAPs in preparation for a public health emergency but also for public health practitioners and policymakers wherever an intervention, policy, or program may not be readily accepted without public education.

## Methods

### Eligibility and recruitment

As this analysis draws upon our earlier work, eligibility and recruitment have been previously described, further detailed here in Supplemental Table 2 [8]. Briefly, we enrolled adults aged 18 or older between May and September 2020 using internet-based snowball sampling in partnership with community patient and health care professional organizations. Additionally, social media sites, including Twitter (currently known as X), Facebook, LinkedIn, and Doximity, were used to recruit participants. Upon enrollment, participants provided informed consent and completed a baseline survey discussing health care disruptions during the pandemic and their opinions and values surrounding scarce resource allocation policies with the framing that such policies may be implemented if pandemic-related surges in hospital utilization reached a breaking point. Surveys were translated (International Contact, Berkeley, CA) and available in English, Spanish, simplified Chinese, Korean, Tagalog, and Vietnamese, California's top 6 spoken languages. Participants were also informed that they would be invited to subsequent surveys and that some would be randomized to receive an educational intervention between survey administrations. As such, participants who provided informed consent and enrolled in our baseline assessment were invited to participate in follow-up. Follow up was collected between October and December 2020.

### Randomization

California participants were randomized to watch a brief educational video explaining UC SRAP [7] tenets. Non-California respondents were allocated to the control arm. California resident respondents underwent stratified randomization at a 1:1 allocation using the native randomization algorithm in QualtricsXM (Qualtrics, Inc., Provo, UT), stratified by self-reported age group (< 35, 35–55, > 55), gender (female vs. all others), race (white vs. all others) and ethnicity (Hispanic/Latin vs. non-Hispanic/Latin), educational attainment (less than bachelor’s degree vs. all others, and health care professional (HCP) employment status (yes vs. no). Study staff were blinded to allocation until the time of analysis; participants were not blinded as to their randomization group, as during the informed consent they were informed of randomization to an intervention or not.

The rationale for this schema was that the UC SRAP would only affect California residents. Therefore, it would be problematic and even potentially unethical (by causing unnecessary emotional distress in considering a potentially moot policy) to randomize non-California residents to learn about a policy that would not necessarily apply to them. However, since we did not restrict the study and received responses from non-California participants, we included them as an additional control for transparency, allowing us to consider cultural effects within California compared to other locations. Participants answered items regarding their values and preferences related to SRAP principles and implementation at baseline and follow-up with those randomized to intervention who received the video immediately before the second assessment. Control participants did not receive the intervention and proceeded directly to the follow-up survey assessment. Power was calculated post hoc as this is a secondary analysis. When comparing the mean agreement between California control and intervention groups, we calculated our sample of 578 with an allocation ratio of 1.09:1 treatment to control to have 85% power to detect a 0.5% difference-in-differences with a standard deviation of 2%.

### Intervention design

The intervention was an animated 6-min video, which explained ethical frameworks (*e.g.,* saving the most lives possible), logistics underpinning how SRAP would function and their rationale (*e.g.,* blinding of patient identity from decision-makers to reduce bias and promote equity, temporary exemptions for health workers), as well as how health and sociodemographic factors would influence allocation priority [10]. The authors (RGB and LEW) drafted the video script with input from the UC Critical Care Bioethics Working Group and targeted a sixth-grade reading level. The animation was designed by a professional video production studio (WorldWise Production, Los Angeles, CA) with voiceover in English and subtitles available in Spanish, simplified Chinese, Korean, Tagalog, and Vietnamese. This video was housed on a private server and embedded directly into the follow-up survey to prevent instrumentation of the control group. Further details on its design are detailed in Supplemental Table 2.

### Endpoint & measurements

For this post hoc analysis, our endpoint was a change in participants’ agreement scores, which denoted alignment with UC SRAP as drafted. Survey instruments developed for this study have been previously published, including their psychometric properties and validation [8]. Agreement scores were defined as the arithmetic distance between the response for each item on a Likert scale and the point on the scale that matched the concept from UC SRAP. For example, for the tenet “Policies should try to save the most lives possible,” a response of 10 on the 10-point scale would denote 100% agreement, and a score of 1 would be 0% agreement.

Items that evaluated how patient factors would influence allocation were operationalized on a 9-point Likert, with 1 being “Should be much less likely” versus 9, “Should be much more likely to get life support,” with 5 as “Should not one way or the other.” The 9-point Likert Scale was chosen to allow maximum variability in responses. Correspondingly, for items on prioritization factors where a factor would not influence resource allocation, a response of 5 would be 100% agreement, while 1 or 9 would each be 0% agreement. These scores were calculated by item and aggregated into four domain scales by taking the mean participant score per domain at each time point. Overall agreement was tabulated by taking the aggregate mean of the four domains at each time point.

### Missing data

There was a small amount of missing data per item used in this analysis, ranging from 2.8% to 5.9%. Based on prior work where we found no substantive differences in imputed versus complete case analyses [9, 10], we did not fit additional models using imputed data for this secondary analysis.

### Statistical analyses

We conducted an intention-to-treat analysis, collating and pairing responses within participants. To determine whether the intervention changed agreement, we employed a difference-in-differences approach to compare the change in score from baseline to follow-up between randomization groups. This approach allowed the calculation of the average treatment effect (ATE) from the intervention while also accounting for secular changes related to different levels of media attention on the possibility of SRAP implementation during the intensifying COVID- 19 crisis [14–16]. To determine ATE, we employed a fractional probit regression with clustered standard errors at the participant level to model percent agreement. Then, we fit marginal estimates with 95% confidence intervals for California intervention vs control groups. Statistical significance was measured by the Wald Z test with Bonferroni corrected 95% confidence intervals and p-values for multiple comparisons. To explore the potential effect of heterogeneity by sociodemographic variables, we fit stratified models by self-reported race/ethnicity, age, education, health care professional employment status, and political affiliation in the same manner. All analyses were completed in Stata 18.0 (StataCorp, College Station, TX) with a two-tailed alpha of 0.05.

## Results

**Fig. 1:**
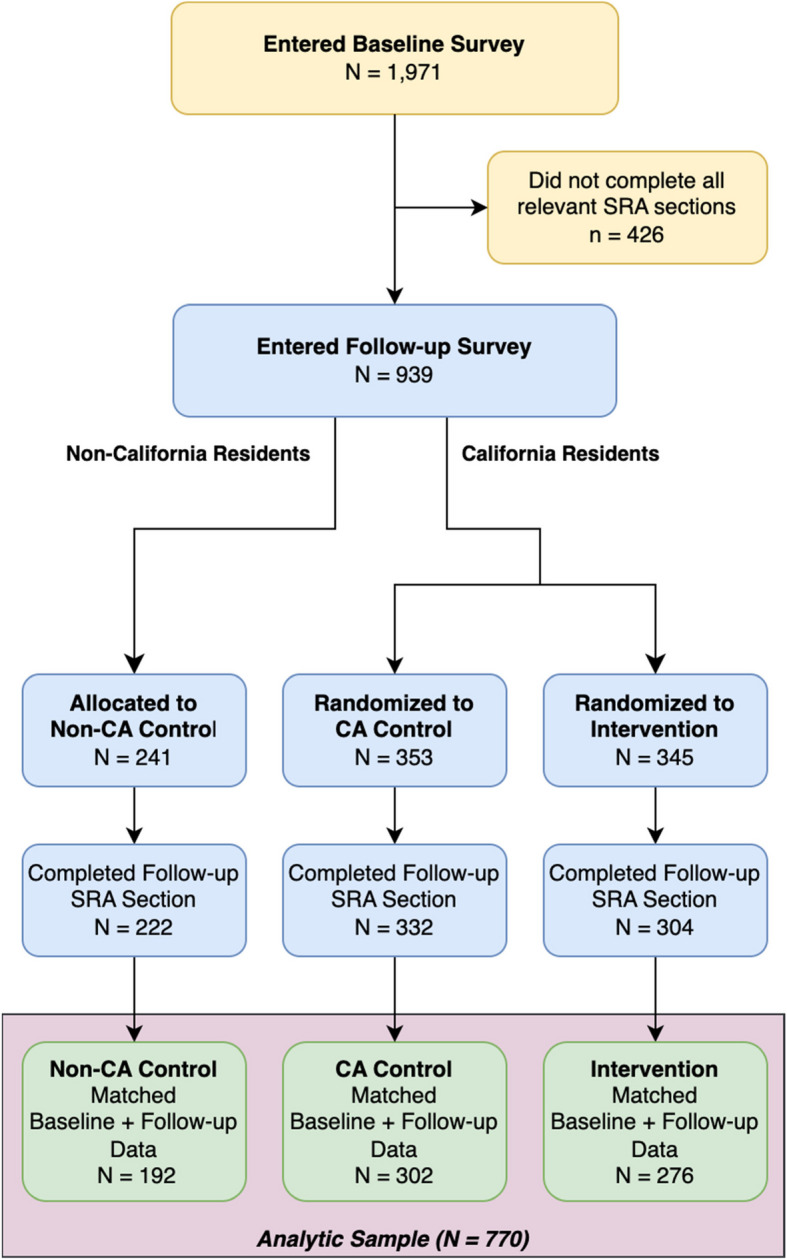
CONSORT diagram demonstrating participant flow and randomization. Alt text: a flow diagram demonstrating how participants were allocated into groups for the study

**Fig. 2:**
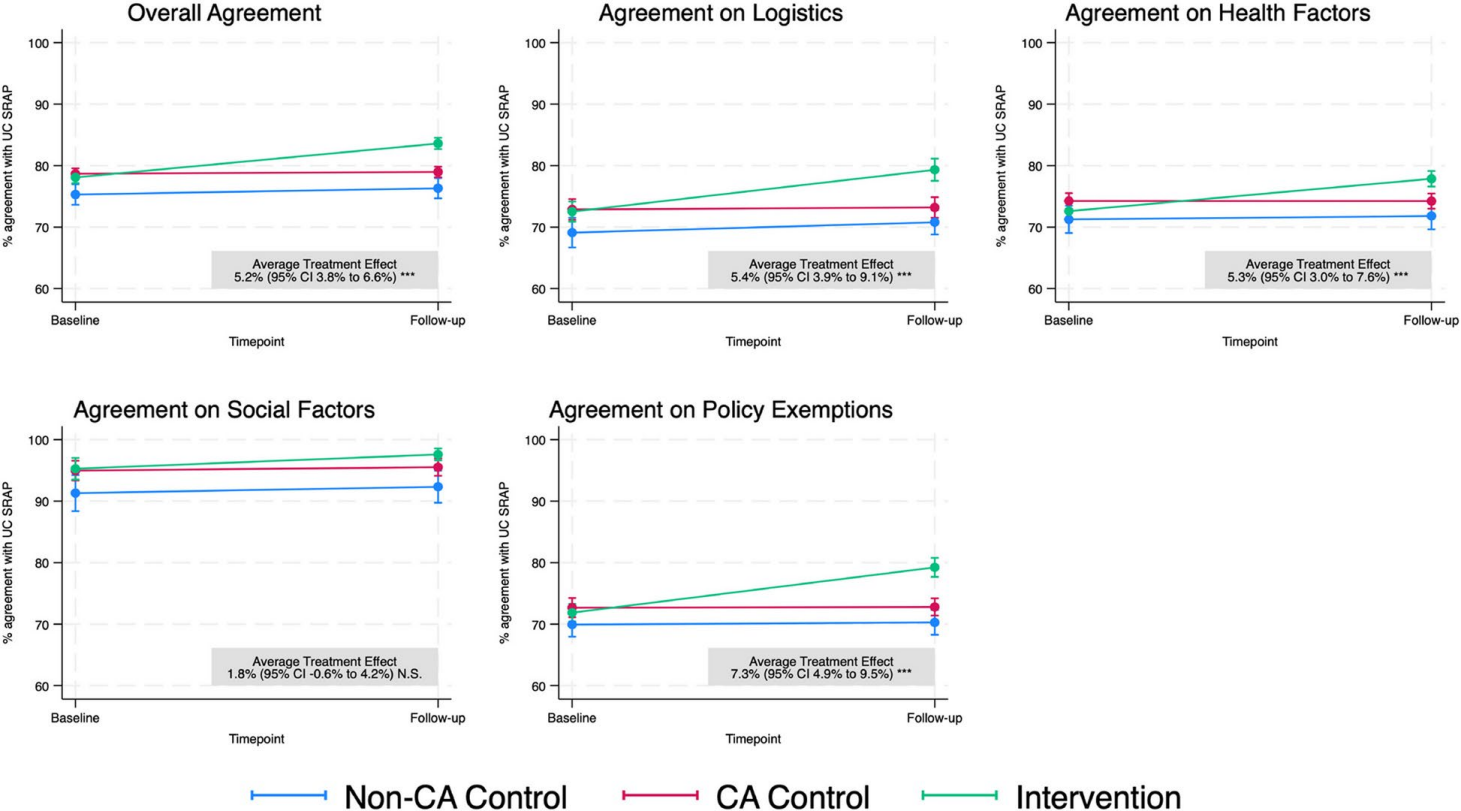
Marginal estimated change in agreement of respondents with UC SRAP tenets by randomization group, overall, and by domain. *P < 0.05, **P < 0.01, ***P < 0.001, N.S = not significant. Alt text: a series of line charts showing estimates of change in agreement with scarce resource policy by randomization group and timepoint

A total of 1,971 adult participants provided informed consent and completed the baseline assessment between May and September 2020. Nine hundred thirty-nine participants completed follow-up assessments between September and December 2020. The time distributions of responses are detailed in Supplemental Figs.1 and 2. Among these participants, 770 individuals had complete baseline and follow-up data and, therefore, were eligible for this analysis (Fig. 1). Participant demographics are shown in Table 1. There were no significant differences in demographics between the randomization groups. Similar to our previously published analyses, we noted high overall baseline agreement across each domain [9]. When we evaluated the effect of viewing the intervention video, we found significant improvements overall and across the domains of policy implementation logistics, health factors considered for allocation, and how exemptions to such policies would apply, as shown in Fig. 2.

**Table 1:** Participant characteristics by group allocation

	All Participants *N* = *770*	Non-CA Control *N* = *192*	CA Control *N* = *302*	CA Intervention *N* = *276*
**Age at enrollment, years, (N) %**				
Younger than 35	(191) 24.8%	(52) 27.1%	(78) 25.8%	(61) 22.1%
35 to 55	(366) 47.5%	(95) 49.5%	(150) 49.7%	(121) 43.8%
Older than 55	(213) 27.7%	(45) 23.4%	(74) 24.5%	(94) 34.1%
**Gender, (N) %**				
Male or other response	(204) 26.5%	(52) 27.1%	(83) 27.5%	(69) 25.0%
Female	(566) 73.5%	(140) 72.9%	(219) 72.5%	(207) 75.0%
Racial/Ethnic Identity*, (N) %				
American Indian/Alaska Native	(13) 1.7%	(2) 1.0%	(6) 2.0%	(5) 1.8%
Asian/Pacific Islander	(93) 12.1%	(27) 14.1%	(34) 11.3%	(32) 11.6%
Black	(42) 5.5%	(15) 7.8%	(15) 5.0%	(12) 4.3%
Hispanic Ethnicity	(94) 12.2%	(21) 10.9%	(39) 12.9%	(34) 12.3%
White	(604) 78.4%	(141) 73.4%	(237) 78.5%	(226) 81.9%
Other race	(20) 2.6%	(7) 3.6%	(6) 2.0%	(7) (2.5%)
**Educational Attainment, (N) %**				
Less than bachelor’s degree	(108) 14.0%	(21) 10.9%	(42) 13.9%	(45) 16.3%
Bachelor's degree or higher	(662) 86.0%	(171) 89.1%	(260) 86.1%	(231) 83.7%
**Health Care Professional, N (%)**	(240) 31.2%	(63) 32.8%	(96) 31.8%	(81) 29.3%
**Political Ideology, (N) %**				
Conservative	(71) 9.2%	(26) 13.5%	(24) 8.0)	(21) 7.6%
Moderate	(117) 15.2%	(30) 15.6%	(44) 14.6)	(43) 15.6%
Liberal	(522) 67.8%	(119) 62.0%	(213) 70.5%	(190) 68.8%
Not political or prefer not to answer	(60) 7.8%	(17) 8.9%	(21) 7.0%	(22) 8.0%

*N.B. *No significant differences were observed by Chi squared tests for any of the above variables

^*^Race/ethnicity was “select all that apply” and may sum to greater than 100%

At baseline, the California control group exhibited 78.7% (95% CI 77.5% to 79.8%) overall agreement, while the intervention group reported 78.1% (76.9% to 79.3%, *P* = 0.36). After the intervention, overall agreement improved by a statistically significantly greater margin of 5.2% (3.8% to 6.6%, *P* < 0.001) for intervention relative to control. Non-California control participants reported consistently lower agreement compared to California control participants: agreement was 3.4% lower agreement at baseline (95% CI 1.5% to 5.3%, *P* < 0.001) and 2.7% lower at follow-up (0.8% to 4.5%, *P* = 0.005).

Agreement with policy logistics improved by 6.5% more in the intervention group, compared to controls (3.9–9.1%, *P* < 0.001), as well as for health factors, with an average treatment effect of 5.3% (3.0%−7.6%, *P* < 0.001). Analyses determined a 7.3% (4.9% to 9.5%, *P* < 0.001) improvement in exemptions to SRAP. However, no significant difference was noted for social factors after intervention. High agreement was measured (> 90%) at baseline for both intervention and control. Agreement per individual item is found in Supplemental Table 1.

In stratified analyses (Table 2), those who self-identified as Asian/Pacific Islander, Hispanic/Latin, or white significantly improved in agreement after the intervention, while those who reported Black, other, or multiracial identity did not. The largest effect was seen in Hispanic/Latin respondents (Supplemental Fig. 3), whose agreement increased by 9.0% (2.9% to 15.1%, *P* = 0.004). There were, otherwise, no substantive differences in the intervention effects by levels of educational attainment (Supplemental Fig. 4), health care professional employment status (Supplemental Fig. 5), or education level (Supplemental Fig. 6). Those reporting conservative political ideology reported a non-significant *decrease* in agreement of − 0.2% (− 5.9% to 5.5%, *P* = 0.94) post-intervention. Those who reported moderate or liberal political ideology both reported similarly significant increases in agreement of 5.8% (2.5% to 9.3%, *P* = 0.001) and 5.7% (4.0 to 7.4%, *P* < 0.001) respectively (Supplemental Fig. 7).

**Table 2:** Average treatment effect of intervention on change in agreement with UC SRAP, stratified by demographics

	Difference-in-Differences/ Average Treatment Effect (95% CI)	Wald Z ***P***-value
**Age, years**		
< 35	4.6% (1.3%, 7.8%)	0.005
35–55	3.9% (1.5%, 6.3%)	0.001
> 55	6.6% (4.5%, 8.6%)	<.001
**Racial/Ethnic Identity**		
Asian/Pacific Islander	6.3% (3.6%, 8.9%)	<.001
Black	5.6% (− 1.5%, 12.7%)	0.12
Hispanic/Latin	9.0% (2.9%, 15.1%)	0.004
More than one race	6.7% (− 9.4%, 22.8%)	0.41
White	4.5% (2.8%, 6.1%)	<.001
Other race	4.5% (− 0.8%, 9.8%)	0.09
**Educational attainment**		
Less than Bachelor's	7.8% (2.8%, 12.9%)	0.002
Bachelor's or higher	4.8% (3.4%, 6.3%)	<.001
**Health care professional employment**		
Non-health care professional	5.8% (4.2%, 7.6%)	<.001
Health care professional	3.4% (1.3%, 6.2%)	0.003
**Political affiliation**		
Conservative	− 0.2% (− 5.9%, 5.5%)	0.94
Moderate	5.8% (2.3%, 9.3%)	0.001
Liberal	5.7% (4.0%, 7.4%)	<.001
Not political or prefer not to answer	5.3% (0.5%, 10.1%)	0.03

## Discussion

In this post hoc analysis of the UC-COVID trial, we found that our educational intervention improved knowledge of the UC SRAP and helped improve community-level agreement with its tenets and principles. Notably, we observed improvements both overall and across stratified subgroup analyses.

Our findings highlight the potential of concise educational interventions to influence public understanding and acceptance of SRAP, a finding extendable to other complex or controversial health care interventions, programs, and policies. These results have important implications for communication with the public, even in the post-pandemic period [17]. While local considerations should be considered whenever feasible, the tenets of the SRAP are rooted in widely accepted medical ethics, which emphasize principles such as fairness, autonomy, beneficence, and justice [2, 3]. As such, not all allocation considerations are negotiable, particularly when they conflict with these foundational principles. Thus, strategies to improve community agreement with these principles are crucial to ensure their successful implementation and adherence. Prior research has explored strategies to foster community agreement, highlighting the importance of transparency, apparent procedural fairness, and adherence to ethical and moral frameworks [18–21]. Consensus building has also been noted to be critical for policy development [19, 22, 23]. However, crises, such as intensive care unit overload during a public health emergency, may limit the ability to perform rigorous community-based participatory research in real-time. This, coupled with the complexity of engaging in partnered research amidst a viral respiratory pandemic with the need for physical distancing, was the rationale for this study. While the use of asynchronous learning or advertising campaigns to improve buy-in to public health campaigns has previously shown mixed effectiveness [24, 25], our findings suggest that combining these strategies with transparent communication has the potential to address barriers to community engagement during times of crisis.

Of note, our analysis identified important demographic differences. For example, we found numerically, although not statistically significantly higher levels of agreement with the SRAP among Californians even at baseline compared to those residing elsewhere. Though the 95% confidence intervals overlapped for this comparison, this suggests potential unmeasured cultural differences that vary across different states and regions that require consideration if these interventions were to be deployed more broadly [26, 27]. We also found heterogeneity in how effective our intervention was at improving agreement across various demographic groups. This highlights the need for additional research to tailor and target messaging to maximize buy-in and ameliorate distrust [28, 29]. For example, while our intervention was available with subtitles in multiple languages, we did not tailor our video to reflect potential cultural differences in key constituent groups. Our findings highlight an area for ongoing research, both in how best to customize messaging for maximal effect and how to balance this with scalability to rapidly deploy an intervention such as this across multiple settings and contexts [29].

Another ongoing question in promoting buy-in of affected parties to policy decisions is the ability to overcome potential entrenchment. Fixed beliefs related to health decisions and public policy can be challenging to move even in the most ideal circumstances. The pandemic has underscored a climate of extreme polarization, with multiple competing interests clashing over how to best balance concerns about mitigating the risk of infection, economic stability, and social cohesion, among other factors. Though prior research has demonstrated the feasibility of many methods in engendering trust in and agreement with health policy decisions [22], these methods are often labor and time-intensive [30]. Our findings suggest that though an asynchronous video learning module cannot wholly overcome a perceived lack of legitimacy or credibility per se, it can assuage some concerns about a lack of transparency or the power imbalance associated with a “top-down” decision. While our study represents the first published intervention of its type in this policy area, replication studies, including multi-site trials or meta-analysis, would improve and validate the long term effectiveness of such interventions.

Further research should continue to explore various communication strategies for disseminating the tenets of an SRAP and rigorously study and disseminate the results of impact analyses of how SRAPs might affect interested parties [31]. Though there are logistical and ethical challenges to empirically studying SRAPs, simulation methods are promising and, for example, can be used to study the impact of the significant state-level variability in how SRAPs are operationalized [6]. There are, however, potential pitfalls and unintended consequences of using simulation methods, including exacerbating health disparities [32–35]. Nevertheless, the results of any empirical evaluation can be used by policymakers to refine SRAPs further and determine how additional data might influence public agreement.

Ultimately, these findings have practical implications for policymakers and healthcare leaders who are tasked with developing and implementing SRAPs during periods of resource scarcity, such as pandemics or public health emergencies. Transparent and accessible communication strategies, such as the educational video used in our intervention, can potentially improve public agreement with its tenets, thus reducing conflicts and improving policy outcomes [1, 20].

### Limitations

Our sampling strategy yielded a sample less diverse (i.e. predominantly female, White) and more highly educated than the overall composition of California [8] limiting generalizability, particularly among historically marginalized groups that may have different perspectives on resource allocation policies. However, our sampling strategy is often acceptable in social science research [36, 37]. Agreement may have been bolstered by social desirability and instrumentation biases, although using control groups will have reduced this to the degree possible. While we did not collect data on reasons for non-completion of surveys nor loss to follow-up, attrition bias should be considered as a potential confounder, as is the case in any longitudinal survey-based study.

## Conclusion

Brief educational interventions provide a robust, transparent tool for improving knowledge about complex health policies and agreement with key ethical principles. We show that even in a policy as ethically complex and politically volatile as determining who should receive the last life-saving critical care resources in a shortage, improvement of agreement with such controversial health services interventions is feasible, acceptable, and effective. This is particularly salient when consensus-building approaches during the promulgation of new policy may not be feasible due to extenuating circumstances. Further research into optimal messaging and dissemination across various constituent groups is needed to build upon this research and improve effectiveness.

## Supplementary Information

Supplementary Material 1.

## Data Availability

Data are avaliable from the authors upon reasonable request and an executed data use agreement with the University of California.

## Abbreviations

RCT: Randomized Controlled Trial
SRAP: Scarce Resource Allocation Policy
UC: University of California
UC-COVID: Understanding Community Considerations, Opinions, Values, and Impacts Study
