# Supplementary material for: Bolstering agreement with scarce resource allocation policy using education: a post hoc analysis of a randomized controlled trial
Source: BMC Health Serv Res. 2025 Apr 14;25:540. doi: 10.1186/s12913-025-12712-x (PMC11995607; doi:10.1186/s12913-025-12712-x)
Supplement: Supplementary file 1 — Supplementary Material 1. [file 12913_2025_12712_MOESM1_ESM.docx]

**Bolstering agreement with scarce resource allocation policy using education: a *post hoc* analysis of a randomized controlled trial**

**Authors & Affiliations:** Russell G. Buhr, MD, PhD^1,2,4^; Cher X. Huang, MD, MSc^1,4^; Ruby Romero, BA^3^; Lauren E. Wisk, PhD^3, 4^

1. Division of Pulmonary & Critical Care Medicine; David Geffen School of Medicine at the University of California, Los Angeles; Los Angeles, CA, USA
2. Center for the Study of Healthcare Innovation, Implementation, and Policy; Health Services Research; Greater Los Angeles Veterans Affairs Healthcare System; Los Angeles, CA, USA
3. Division of General Internal Medicine & Health Services Research; David Geffen School of Medicine at the University of California, Los Angeles; Los Angeles, CA, USA
4. Department of Health Policy & Management; Fielding School of Public Health at the University of California, Los Angeles; Los Angeles, CA, USA

**Supplemental Tables:**

eTable 1: Individual item agreement by time point and intervention group

|  | **Baseline** | | **Follow-up** | | **Difference-in-Differences** | |
| --- | --- | --- | --- | --- | --- | --- |
|  | *CA Control Arm* | *CA Treatment Arm* | *CA Control Arm* | *CA Treatment Arm* | *Tx Effect* | |
| **Item** | *% Agreement* | | *% Agreement* | | *𝚫 % Agree* | *P-value* |
| ***Values - Logistics*** |  |  |  |  |  |  |
| Try to save the most number of lives possible | 89.2% | 91.4% | 90.1% | 91.1% | -2.0% | 0.33 |
| Take life support away from some patients to give to others | 52.3% | 50.8% | 49.3% | 53.3% | 5.6% | 0.04 |
| Make decisions on a first-come, first-served basis | 70.7% | 72.9% | 64.6% | 77.2% | 10.3% | <.001 |
| Apply the same rules to all patients equally | 76.8% | 76.6% | 78.3% | 84.8% | 6.7% | 0.03 |
| Same rules even if pt admitted before the crisis | 74.6% | 74.2% | 79.0% | 80.5% | 1.8% | 0.53 |
| Same rules even if pt hospitalized unrelated to pandemic | 75.3% | 76.2% | 79.8% | 83.2% | 2.5% | 0.87 |
| Hospital committees should make these decisions | 57.1% | 54.1% | 55.0% | 75.0% | 23.0% | <.001 |
| Hospital committees should not know pt identities | 84.8% | 83.3% | 84.6% | 89.9% | 6.7% | 0.006 |
| **Total Agreement - Logistics** | 72.9% | 72.5% | 73.2% | 79.3% | 6.5% | <.001 |
| ***Values - Health Factors*** |  |  |  |  |  |  |
| Patients who are deemed less likely to survive | 73.4% | 69.5% | 67.8% | 75.0% | 11.1% | <.001 |
| Patients who have physical or intellectual disabilities | 91.8% | 91.8% | 93.4% | 96.8% | 3.4% | 0.13 |
| Patients with shorter expected lifespans b/c chronic illness | 60.6% | 60.0% | 59.1% | 61.7% | 3.2% | 0.03 |
| Patients who are elderly | 80.3% | 78.4% | 84.3% | 86.1% | 3.6% | 0.17 |
| Patients expected to have a poor quality of life if they survive | 64.7% | 62.4% | 66.6% | 69.7% | 5.4% | 0.06 |
| **Total Agreement - Health** | 74.3% | 72.6% | 74.2% | 77.9% | 5.3% | <.001 |
| ***Values - Social Factors*** |  |  |  |  |  |  |
| People who are wealthy, famous, or in positions of power | 96.0% | 96.3% | 96.1% | 98.5% | 2.1% | 0.16 |
| People who are a racial or ethnic minority | 96.5% | 95.7% | 96.8% | 98.2% | 2.2% | 0.16 |
| People who are LGBTQ+ | 97.2% | 96.1% | 97.4% | 99.4% | 3.2% | 0.04 |
| People who are prisoners | 90.2% | 91.7% | 91.0% | 93.7% | 1.2% | 0.54 |
| People without health insurance | 96.2% | 96.3% | 97.2% | 98.7% | 1.3% | 0.35 |
| People who are undocumented immigrants | 94.0% | 95.2% | 95.1% | 97.1% | 0.9% | 0.60 |
| People w/ shorter lifespan even if mostly disabled | 81.5% | 79.3% | 84.5% | 81.8% | -0.5% | 0.84 |
| People w/ shorter lifespan even if mostly minority | 82.1% | 80.5% | 84.9% | 81.6% | 1.8% | 0.47 |
| **Total Agreement - Social** | 95.0% | 95.3% | 95.5% | 97.6% | 1.8% | 0.16 |
| ***Values - Exemptions*** |  |  |  |  |  |  |
| Patients who are pregnant in the first trimester | 64.7% | 61.3% | 68.7% | 72.2% | 6.9% | 0.05 |
| Patients who are pregnant in the third trimester | 79.6% | 78.9% | 78.1% | 83.6% | 6.3% | <.001 |
| First responders | 67.8% | 68.3% | 68.7% | 75.1% | 5.8% | 0.002 |
| Health care workers in general | 72.8% | 73.6% | 72.4% | 80.1% | 6.9% | <.001 |
| Health care workers specifically on the front lines | 77.2% | 77.2% | 76.2% | 85.2% | 9.0% | <.001 |
| **Total Agreement - Exemptions** | 72.6% | 71.8% | 72.8% | 79.2% | 7.3% | <.001 |
| **Overall Total Agreement** | 78.7% | 78.1% | 79.0% | 83.6% | 5.2% | <.001 |

*eTable 2: Eligibility, recruitment, and intervention design summary*

| **Item** |  |
| --- | --- |
| ***Recruitment*** |  |
| Target Population | California residents, enriched for health care professionals; participation open to non-California residents but recruitment not directly targeted to them. |
| Recruitment Strategy | Email, social media (including professional language targeted posts on social media sites (e.g., Doximity, LinkedIn) to recruit a sample of health care providers), and snowball sampling. |
| ***Eligibility*** |  |
| Inclusion Criteria | Adults 18+ who are competent to provide informed consent. Detailed description of participants was previously published [1] |
| Exclusion Criteria | Not applicable – any adult 18 years or older was eligible. |
| ***Intervention Design*** |  |
| Duration | 6-minutes |
| Purpose | Educational “explainer” video covering the mechanics and ethical principles underpinning the UC SRAP. |
| Randomization | California residents were randomized using a parallel randomization algorithm (Qualtrics XM) at a 1:1 ratio as stratified by self-reported gender (female compared with all others), health care  professional (HCP) occupation, education (<Bachelor’s degree compared with ≥Bachelor’s degree), race (American Indian/Alaska Native, Asian/Pacific Islander, and Black individuals compared with  White individuals), Hispanic ethnicity, and age (<35, 35-55, and >55 years). Intervention participants viewed the brief video and completed follow-up survey items; control participants proceeded  directly to the follow-up without the video. |
| Animation/Translation | A script was drafted by the authors (RGB, LEW) summarizing the tenets in the UC SRAP with feedback from the UC Critical Care Bioethics Working Group at a sixth grade reading level and furnished to a production studio (WorldWise Production) who animated it with a voiceover in English and subtitles in Spanish, simplified Chinese, Vietnamese, Korean, and Tagalog (the top 5 non-English languages spoken in California. International Contact translated the video. The video introduced the SRAP, outlined the circumstances of its use, and explained the ethical principles and rationale underpinning the policy, the logistics of how the SRAP would function, consequences of non-allocation, and patients’ rights. The video was previously published as a supplement to another manuscript [2], and is available online at <https://jamanetwork.com/journals/jama-health-forum/fullarticle/2824903#multimedia-tab> |

**Supplemental Figures:**


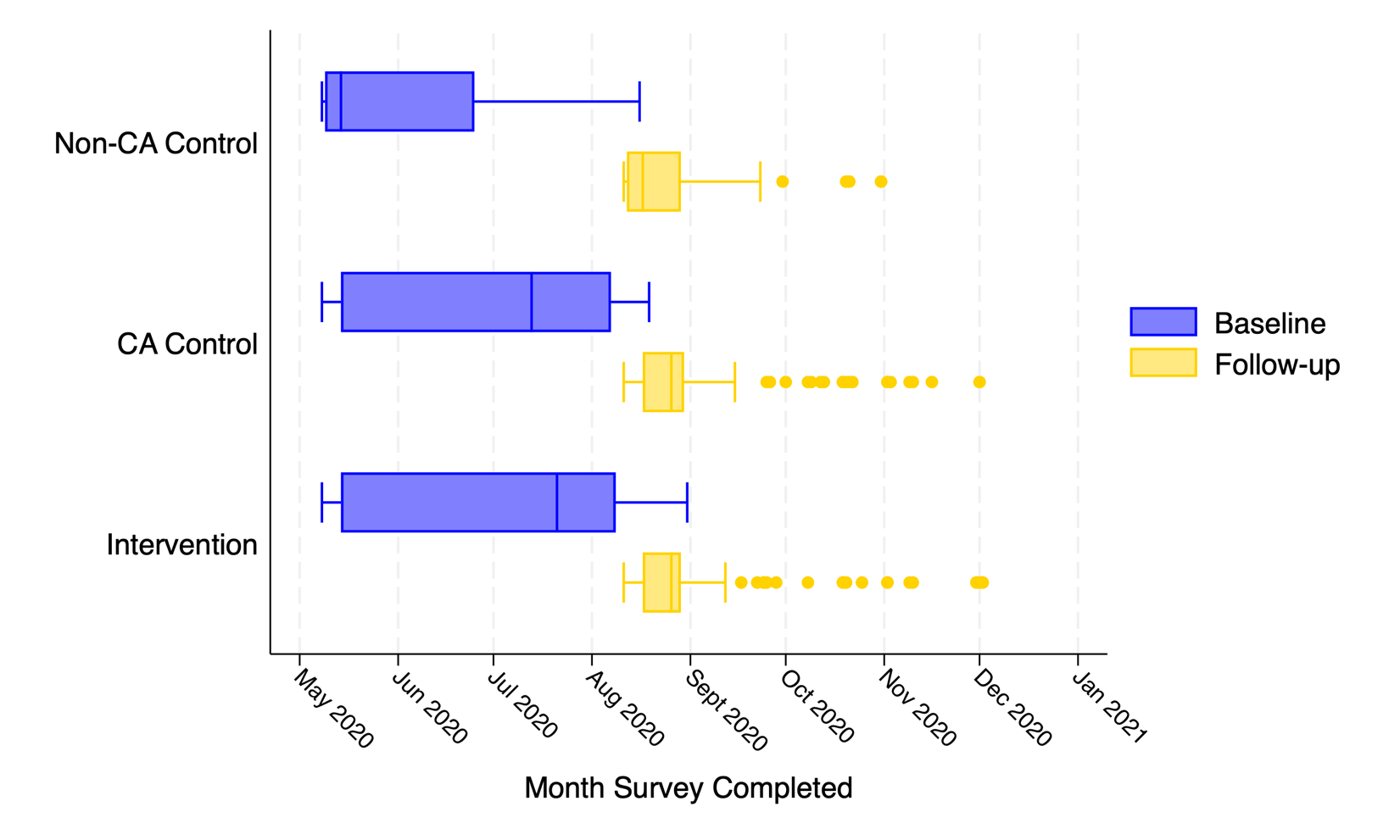


Supplemental Figure 1: Timing of survey administrations by randomization group

*Alt text: box plots showing when participants enrolled in the study and completed follow up, stratified by randomization group*


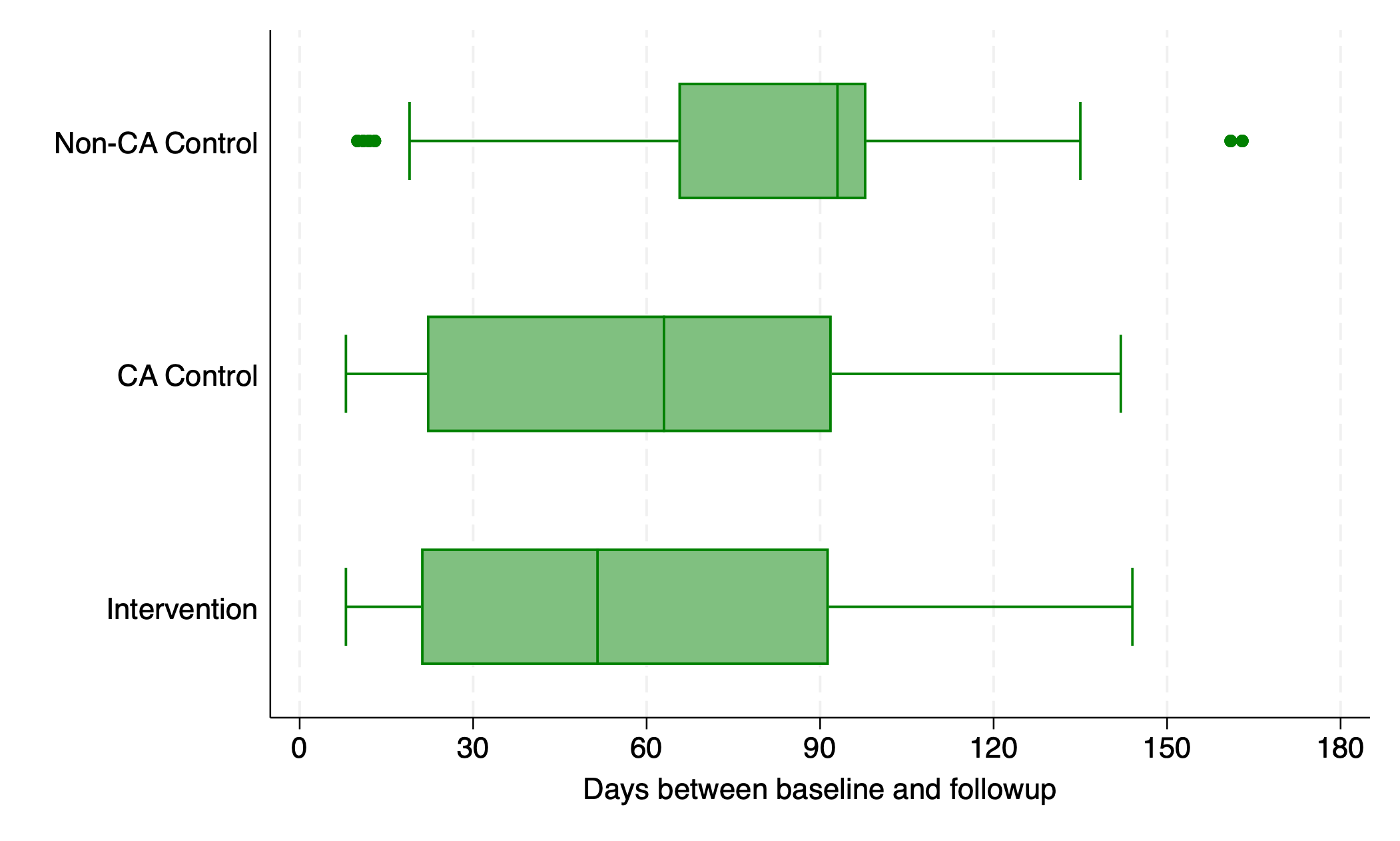


Supplemental Figure 2: Distribution of time between survey administrations by randomization group

*Alt text: box plots showing when participants elapsed time between enrollment and follow up, stratified by randomization group*


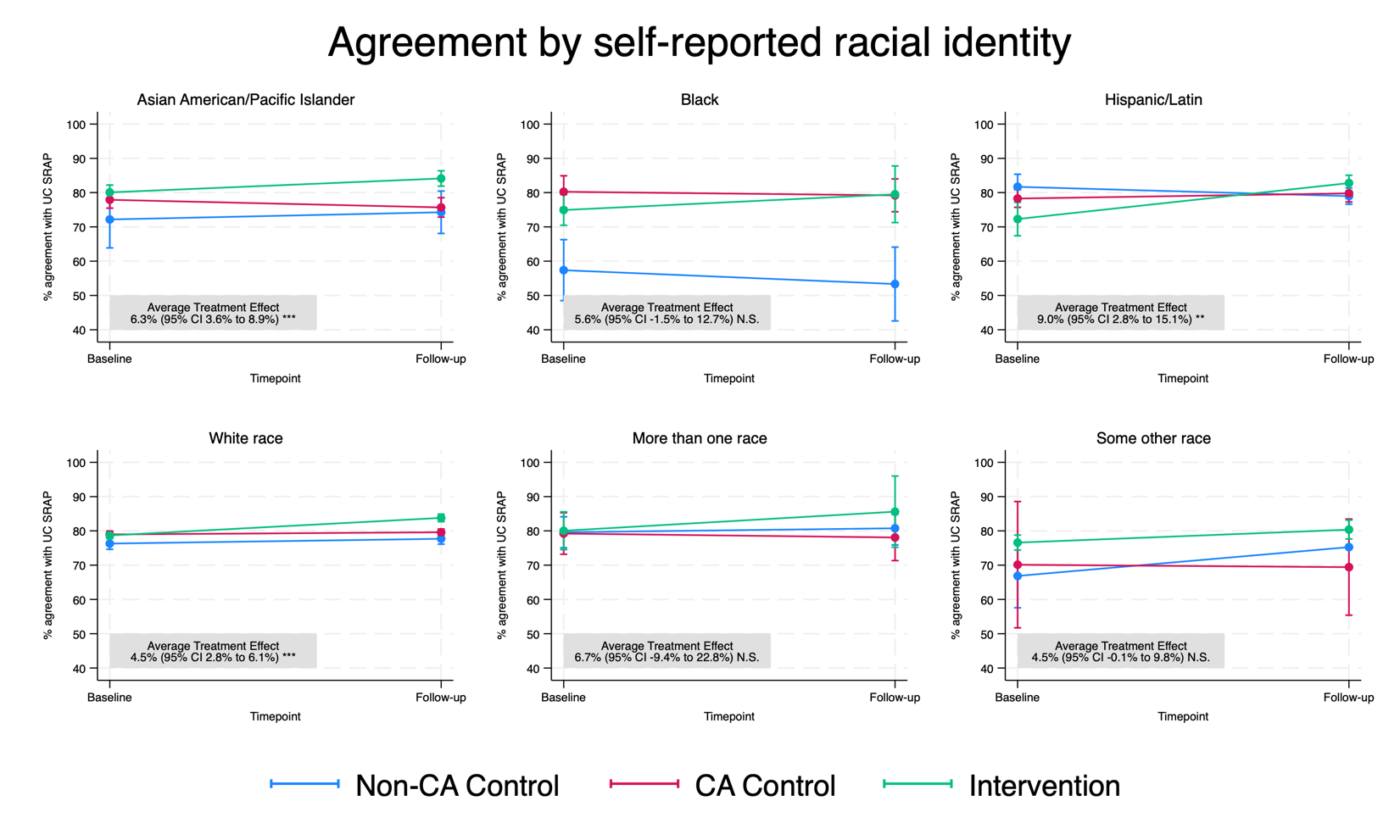


Supplemental Figure 3: Agreement stratified by self-identified race/ethnicity

N.B.: *** denotes P<.001, ** P <.01, * P<.05, N.S. not significant

*Alt text: a series of line charts showing change in agreement with scarce resource policy by randomization group and timepoint, stratified by race and ethnicity*


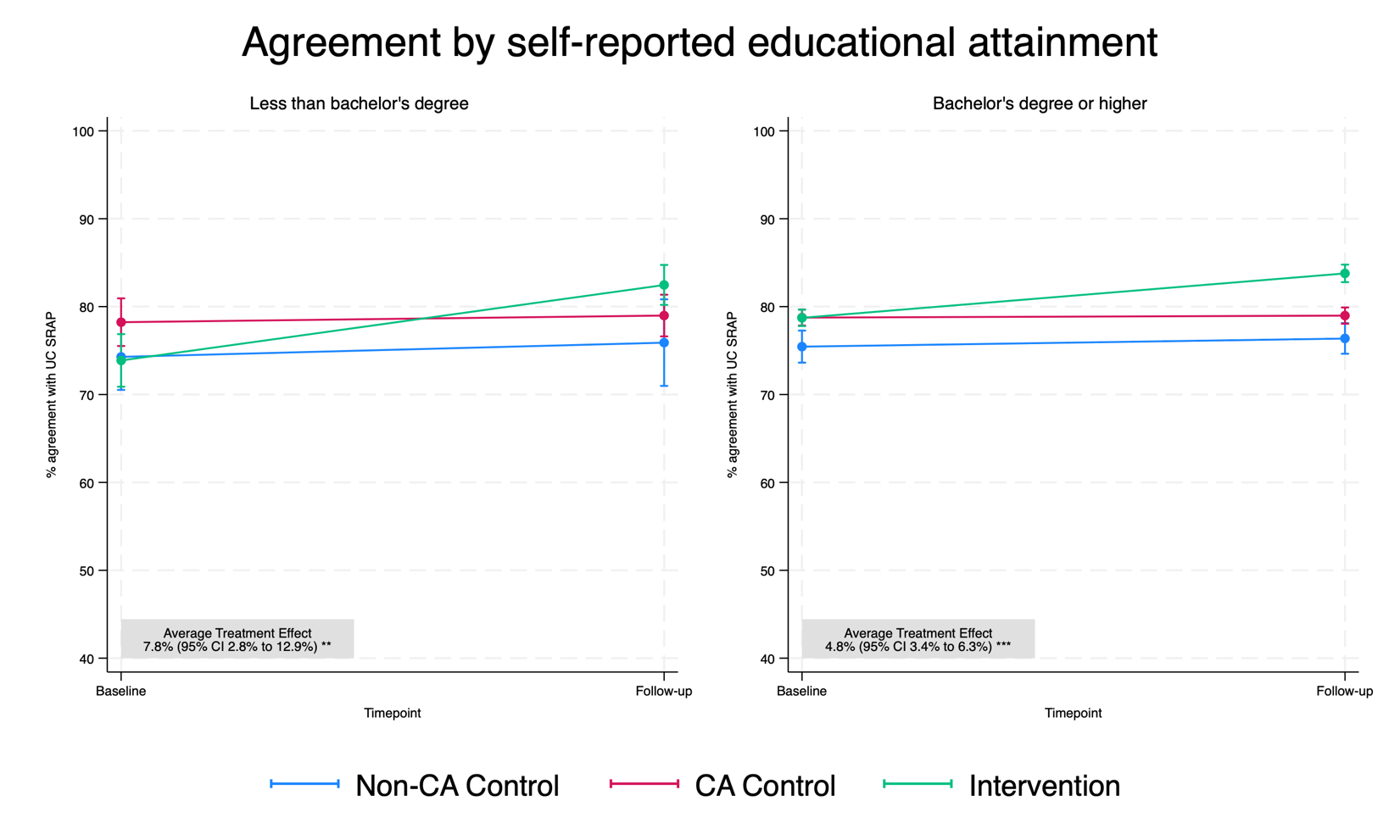


Supplemental Figure 4: Agreement stratified by self-identified educational attainment

N.B.: *** denotes P<.001, ** P <.01, * P<.05, N.S. not significant

*Alt text: a series of line charts showing change in agreement with scarce resource policy by randomization group and timepoint, stratified by educational attainment*


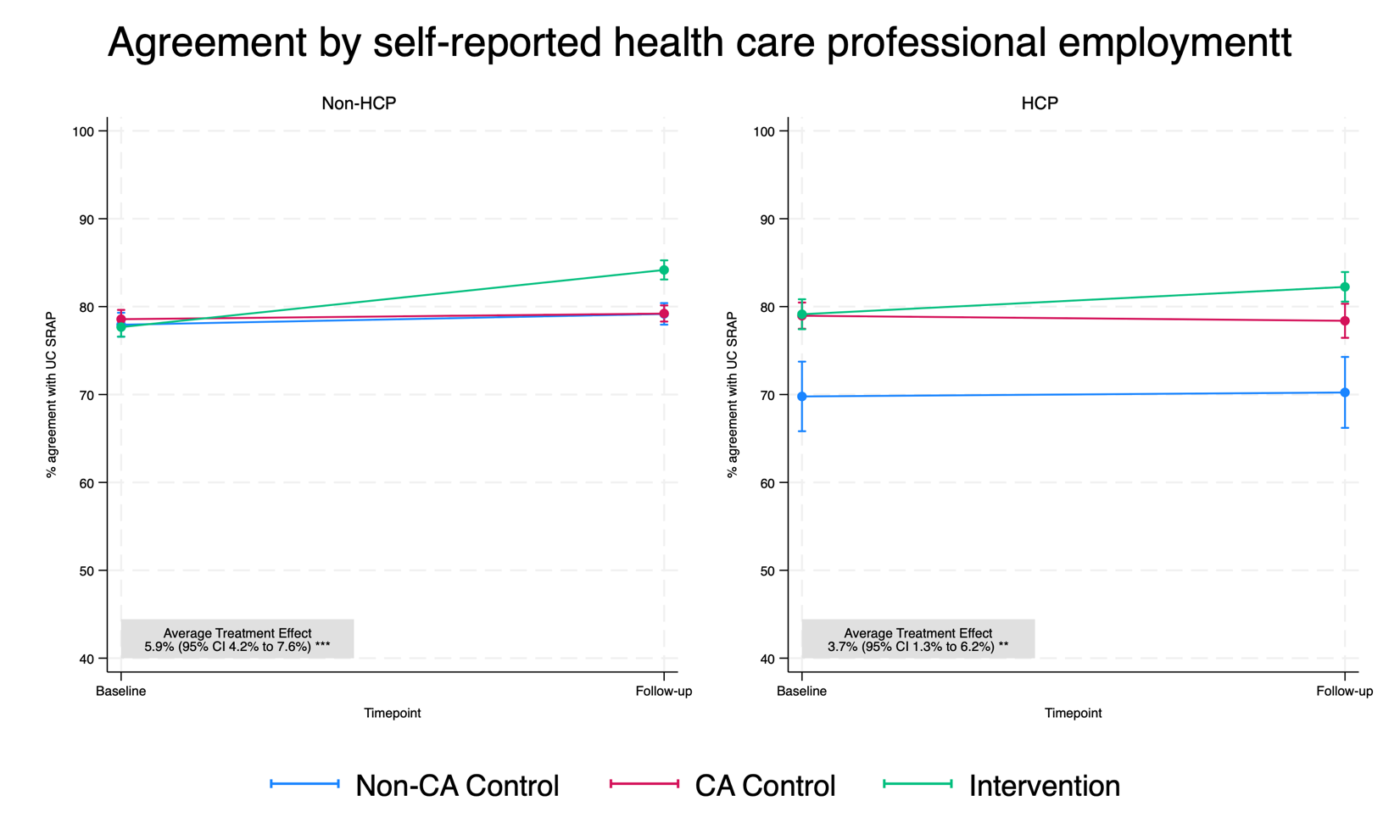


Supplemental Figure 5: Agreement stratified by self-identified health care professional employment status

N.B.: *** denotes P<.001, ** P <.01, * P<.05, N.S. not significant

*Alt text: a series of line charts showing change in agreement with scarce resource policy by randomization group and timepoint, stratified by health care professional employment status*


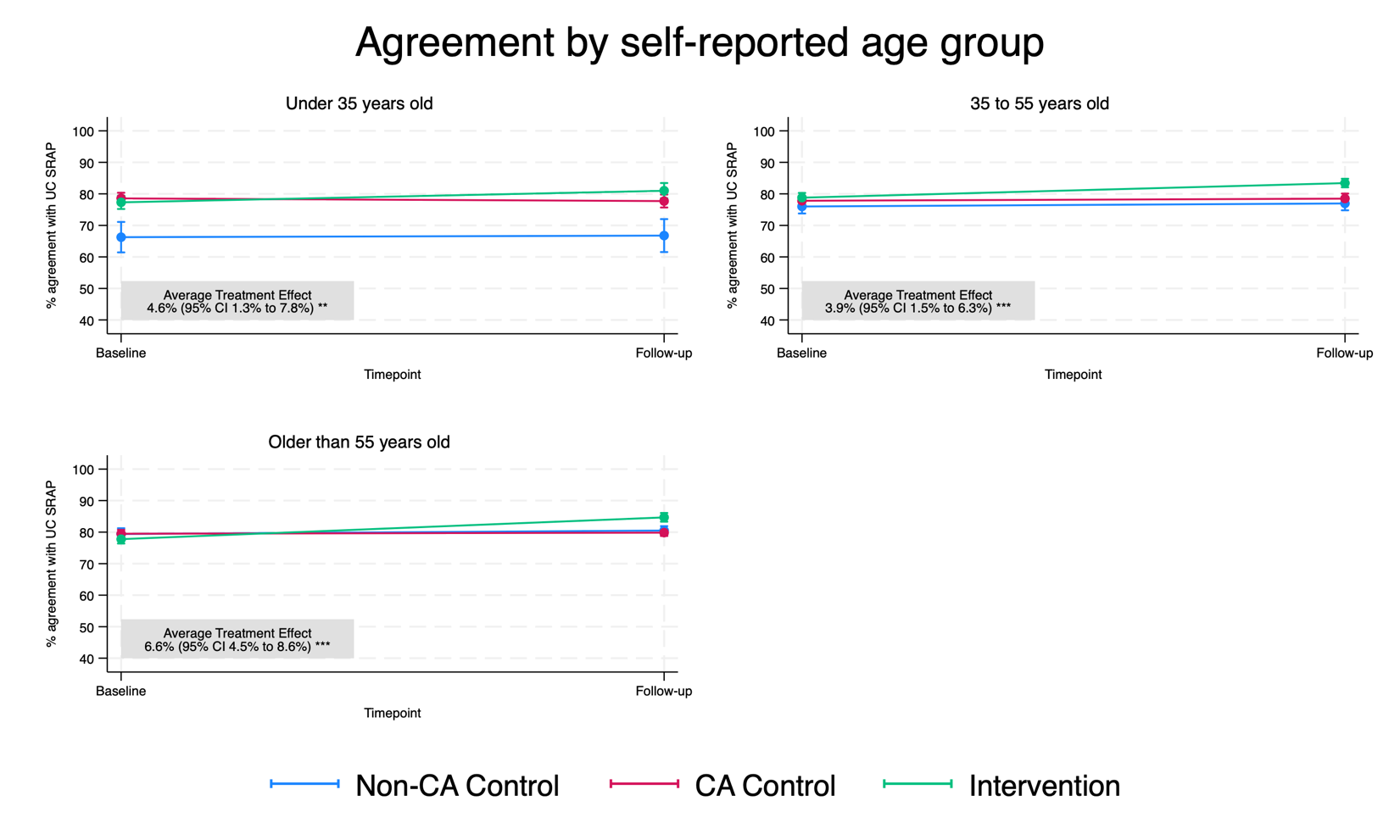


Supplemental Figure 6: Agreement stratified by self-identified age group

N.B.: *** denotes P<.001, ** P <.01, * P<.05, N.S. not significant

*Alt text: a series of line charts showing change in agreement with scarce resource policy by randomization group and timepoint, stratified by age group*


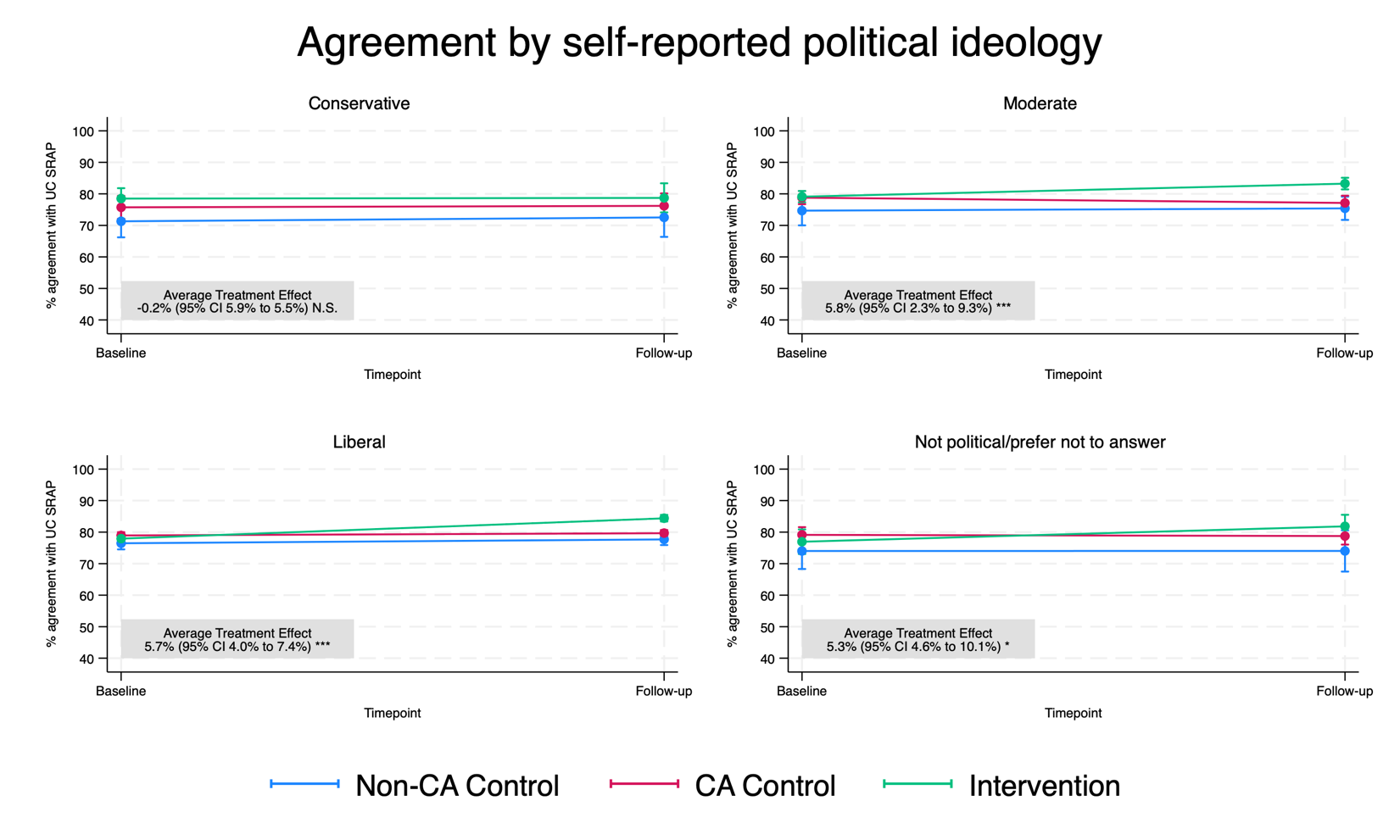


Supplemental Figure 7: Agreement stratified by self-identified political ideology

N.B.: *** denotes P<.001, ** P <.01, * P<.05, N.S. not significant

*Alt text: a series of line charts showing change in agreement with scarce resource policy by randomization group and timepoint, stratified by political ideology*

Bibliography & References Cited

1. Wisk LE, Buhr RG: **Rapid deployment of a community engagement study and educational trial via social media: implementation of the UC-COVID study**. *Trials* 2021, **22**(1):513.

2. Buhr RG, Romero R, Wisk LE: **Promoting knowledge and trust surrounding scarce resource allocation policy: a randomized controlled educational intervention trial**. *JAMA Health Forum* 2024, **5**(10):e243509.
